# The Potential Cerebrovascular Sequelae of Coronavirus Disease 19 in Alzheimer’s Disease: Coincidental or Causative?

**DOI:** 10.5152/eurasianjmed.2022.21146

**Published:** 2022-02-01

**Authors:** Abdullah Egiz, Sarvin Farajzadeh-Asl, Bassel Alaa Abouzeid, Radhi Al-Shaharli, Rand Al-Assadi

**Affiliations:** 1 University of Central Lancashire School of Medicine, Preston, United Kingdom; 2 Department of Neurology, Royal Blackburn Teaching Hospital, Blackburn, United Kingdom

Dear Editor,

Since coronavirus disease 2019 (COVID-19) was declared a pandemic by the World Health Organization on March 11, 2020, the impact of this global health crisis has resulted in numerous disruptions to clinical research.^1^ Coronavirus disease 2019 seems to be leaving a long-lasting effect, reducing the quality of life of many individuals.^1^ The current research suggests that the long-term sequelae of COVID-19 are likely to disproportionately affect high-risk individuals including Alzheimer’s disease (AD) patients.^1^ The neuropsychiatric sequelae of COVID-19 have gained attention given its projected weight in the global burden of disability.^1^ However, it seems that a deeper look into the effect of COVID-19 on AD has overlooked the potential cerebrovascular alterations. Therefore, we would like to highlight the potential cerebrovascular sequelae associated with COVID-19 infections in patients with AD as shown in Figure 1.

Alzheimer’s disease and dementia are an increasing global health challenge, affecting nearly 40-50 million people.^2^ Alzheimer’s disease is a neurodegenerative disease caused by the formation of β-amyloid plaques and neurofibrillary tangles in the cortex, manifesting clinically as a cognitive impairment that progressively declines with age.^2^ Given the prevalence of the disease, many individuals are at risk of acquiring COVID-19 infections and possibly experiencing neurological sequelae.^1^ A systematic review by Fraiman et al^3^ concluded that cerebrovascular events are common findings in COVID-19 infections as authors’ analyses reported 275 patients experienced cerebrovascular accidents (CVAs), ischemic stroke, intracranial hemorrhage, and cerebral venous sinus thrombosis.^3,4^

Prior to COVID-19 era, cerebrovascular alterations were proven in AD.^4^ Iadecola and Gottesman^4^ concluded that although vascular and neurodegenerative changes are distinct, yet they are intertwined substrates of age-related cognitive impairment.^4^ Among the proposed hypotheses for the mechanisms of cerebrovascular alterations, caused by COVID-19, is the viral entry, into endothelial cells, via the angiotensin-converting enzyme 2 (ACE-2) receptors. The ACE-2 receptors on brain capillary endothelial cells are known to allow COVID-19 viral invasion of the cells, which may trigger neuroinflammation. The neuroinflammation triggers the release of interleukin-6 (IL-6), IL-10, and tumor necrosis factor-alpha, eventually precipitating a cytokine storm.^5^ Tumor necrosis factor-alpha, which can cross the blood-brain barrier),^5^ is known to activate microglia and astrocytes, causing even more inflammation.^5^ A severe COVID-19 infection associated with a cytokine storm can potentially cause endothelial damage, leading to an increased risk of CVAs.^3-4^ The damage of COVID-19 to endothelial cells was found to increase the risk of micro-thrombus formation due to the upregulation of von Willebrand factor, causing fibrin deposition, platelet adhesion, and activation, causing more inflammation and vascular complications.^3-5^ Although the exact mechanism remains unknown, the effects of COVID-19 on the brain may potentially cause cerebrovascular sequelae with or without exacerbation of neurocognitive function. Nevertheless, various reports have described hypercoagulability associated with severe COVID-19 illness due to immobilization, inflammation, endothelial cell injury, and platelet activation,^3-5^ further adding to the risk of developing CVAs.^3-5^

In summary, severe COVID-19 infections may cause potential cerebrovascular complications in patients with AD. The proposed mechanism escalates the question of whether exploring the normalization of endothelial cells through novel or repurposed prophylactic drugs would prevent unwanted vascular-related COVID-19 sequelae.

## Figures and Tables

**Figure 1.**
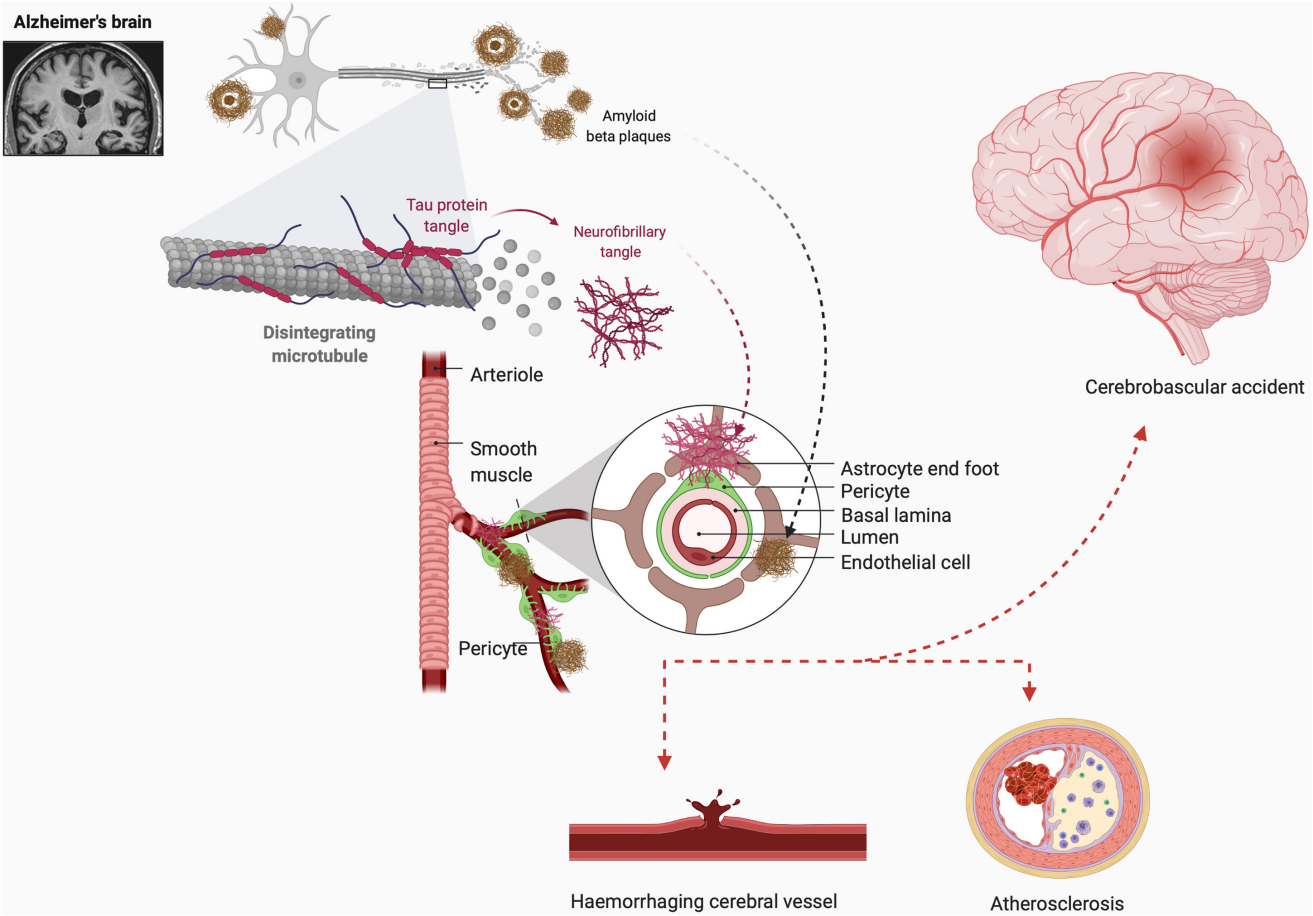
The pathological changes in Alzheimer’s disease lead to cerebrovascular alterations by causing vascular dysregulation, blood-brain barrier disruption, and promoting atherosclerosis. Created with BioRender.com.
